# Cavitary pulmonary tuberculosis with COVID-19 coinfection

**DOI:** 10.1016/j.idcr.2020.e00973

**Published:** 2020-09-28

**Authors:** Zohaib Yousaf, Adeel A. Khan, Haseeb A. Chaudhary, Kamran Mushtaq, Jabeed Parengal, Mohamad Aboukamar, Muhammad Umair Khan, Mouhand F.H. Mohamed

**Affiliations:** a Department of Medicine, Hamad Medical Corporation, Doha, Qatar; b Dresden International University, Dresden, (DIU), Germany; c Medicine, Reading Hospital, Tower Health Medical Group, West Reading, United States; d Department of Gastroenterology and Hepatology, Hamad Medical Corporation, Doha, Qatar; e Department of Infectious Disease, Hamad Medical Corporation, Qatar

**Keywords:** SAR-CoV-2, COVID-19, Mycobacterium tuberculosis, TB, Co-infection, Pulmonary tuberculosis

## Abstract

The COVID-19 pandemic has strained the healthcare system worldwide, leading to an approach favoring judicious resource allocation. A focus on resource preservation can result in anchoring bias and missed concurrent diagnosis. Coinfection *of Mycobacterium tuberculosis* (TB) and severe acute respiratory syndrome coronavirus 2 (SARS-CoV-2) has implications beyond morbidity at the individual level and can lead to unintended TB exposure to others. We present six cases of COVID-19 with newly diagnosed cavitating pulmonary tuberculosis to highlight the significance of this phenomenon and favorable outcomes if recognized early.

## Background

The modern-day medical practice uses William Osler’s principles of clinical reasoning. “Occam’s razor” advocates a simplistic approach where all symptoms are attributed to a single disease [1]. The counterargument to this diagnostic parsimony is “Hickam's dictum," which favors disease plurality in a single patient. Numerous shortcomings in the current COVID-19 pandemic have skewed our practice in favor of the former principle to preserve resources. This practice has led to anchoring bias and overlooking of other concomitant diseases [2]. Historical evidence suggests higher mortality in patients with concurrent infection of TB and respiratory viruses, such as influenza [3,4]. We present six COVID-19 patients who were co-infected with pulmonary TB with a favorable outcome. All patients had an initial working diagnosis of COVID-19 pneumonia on triage. It was on a detailed review that the probability of pulmonary TB coinfection became apparent leading to isolation from other COVID-19 patients.

## Case presentations

### Subjects

We performed a retrospective analysis of the six male patients co-infected with TB and COVID-19 (software utilized: Jamovi version 1.1.9, available at www.jamovi.org). Extracted data included baseline demographics, clinical characteristics, laboratory values, imaging, and microbiologic findings.

### Patient characteristics

All patients were male owing to the preponderance of male immigrant population in Qatar. The mean age of patients was 35.5 years. All patients were from TB endemic area of south-east Asia. Only one patient had previous diagnosis of diabetes mellitus (DM). Other patients had no previous co-morbidities. The mean duration of symptoms prior to hospitalization was 14 days. Detailed information is shown in Table 1.

**Table 1:** Patient’s characteristics.

Characteristics	Patient 1	Patient 2	Patient 3	Patient 4	Patient 5	Patient 6
Age	34	32	50	35	27	35
Sex	Male	Male	Male	Male	Male	Male
Nationality	Nepalese	Nepalese	Indian	Bangladeshi	Indian	Bangladeshi
Past Medical History	None	None	Diabetes Mellites	None	None	None
Duration of symptoms (days)	10	8	21	30	5	10
Symptoms	Fever, productive cough, myalgias	Dry cough, fatigue.	Dry cough	dry cough, fever	Fever, myalgias, headache	Dry cough
B-Type symptoms	Subjective weight loss	5 % Weight loss over 6 months	Subjective weight loss	5 % weight loss over 3months	None	Subjective weight loss

### Symptomatology and lab results

**Fig. 1:**
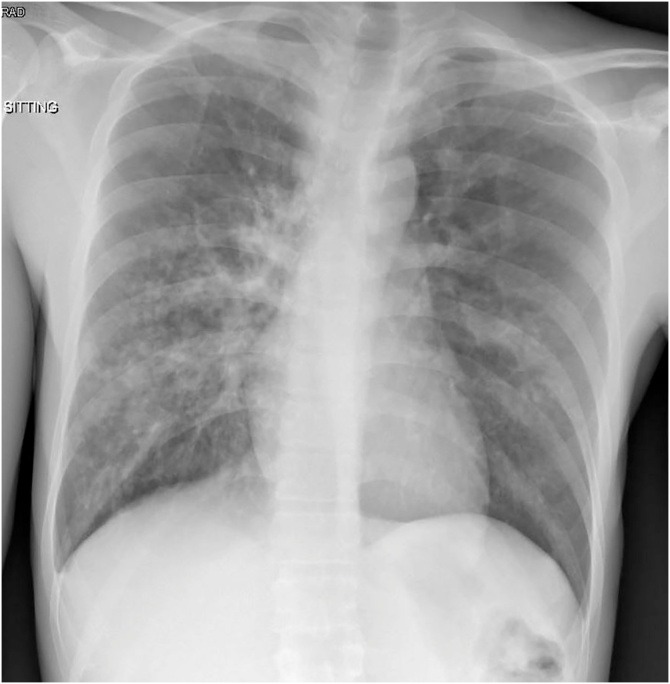
Bilateral infiltrates with multiple cavitary lesions.

**Fig. 2:**
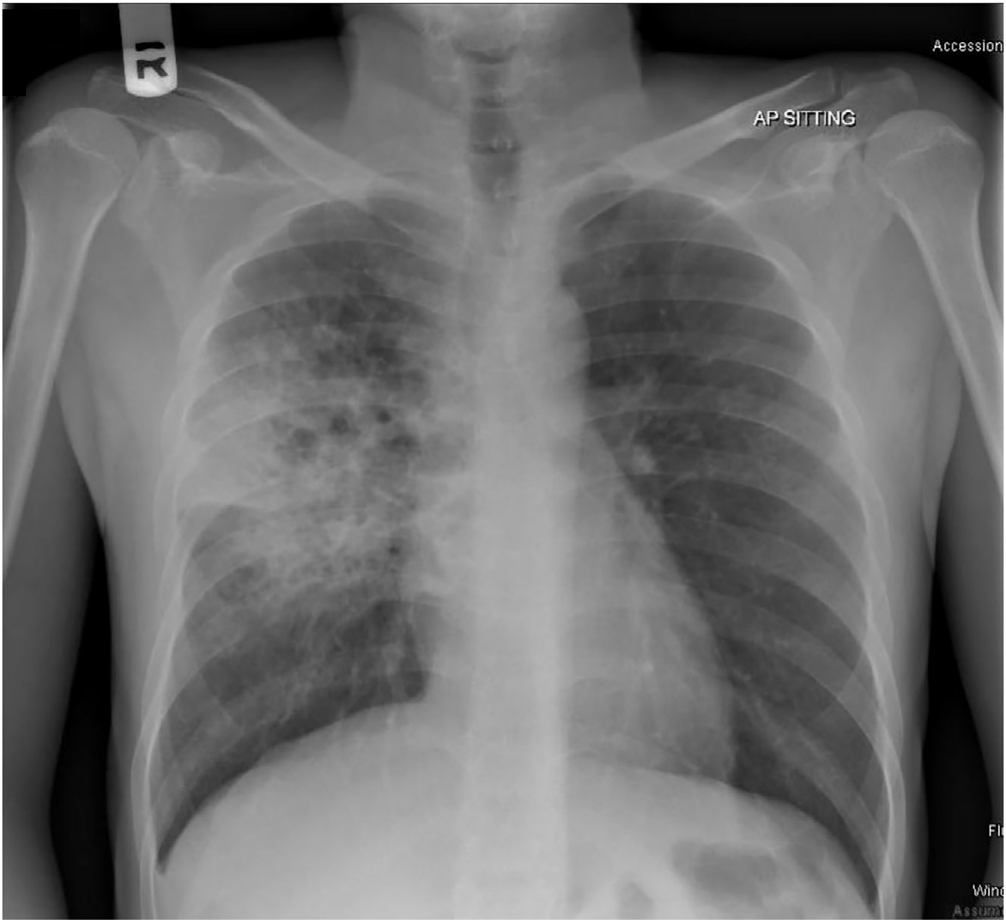
Predominantly right middle zone infiltrates with multiple cavitary lesions.

**Fig. 3:**
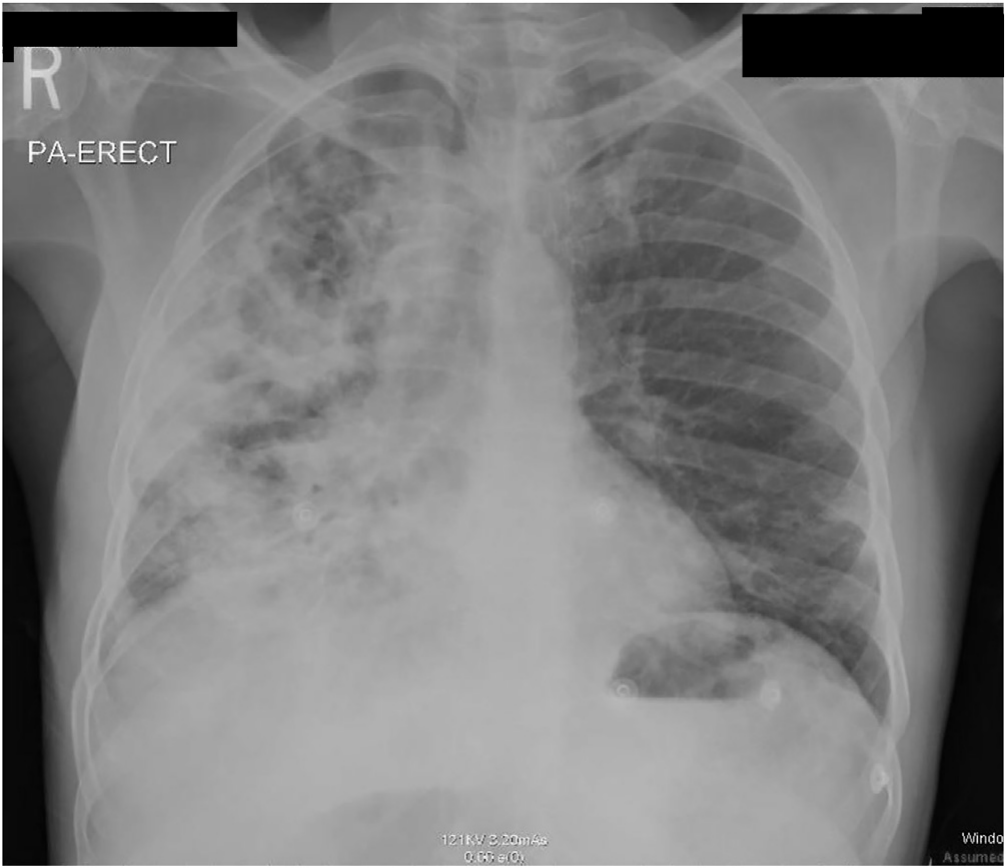
Bilateral infiltrates with extensive involvement on right side with multiple cavitary lesions.

**Fig. 4:**
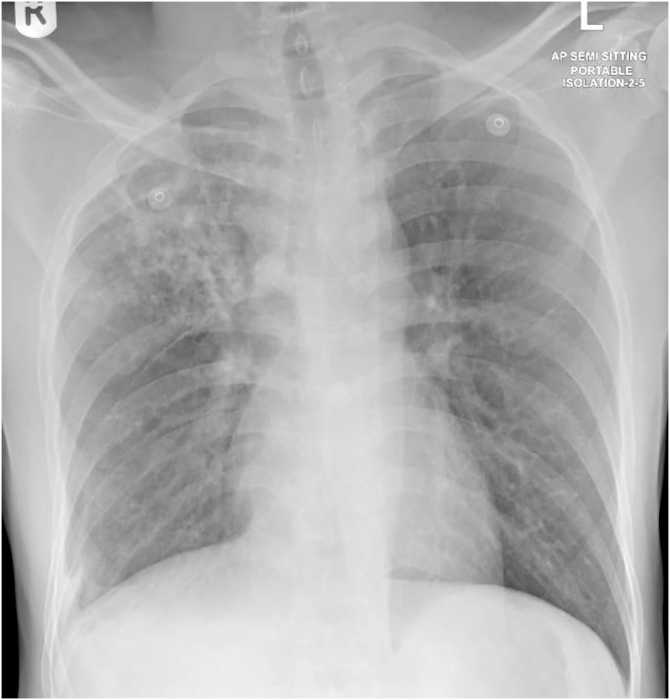
Bilateral infiltrates, more prominent in right upper zone with multiple cavitary lesions.

**Fig. 5:**
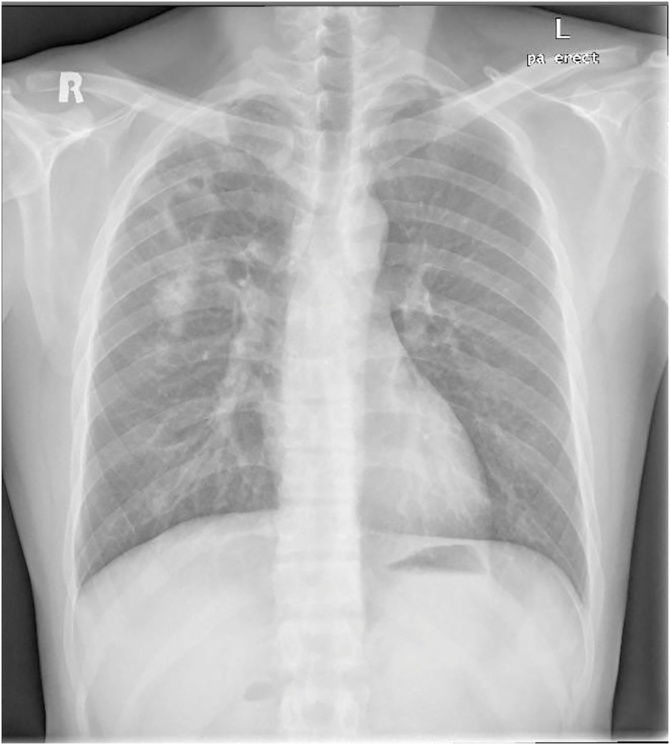
Bilateral infiltrates with multiple cavitary lesion.

**Fig. 6:**
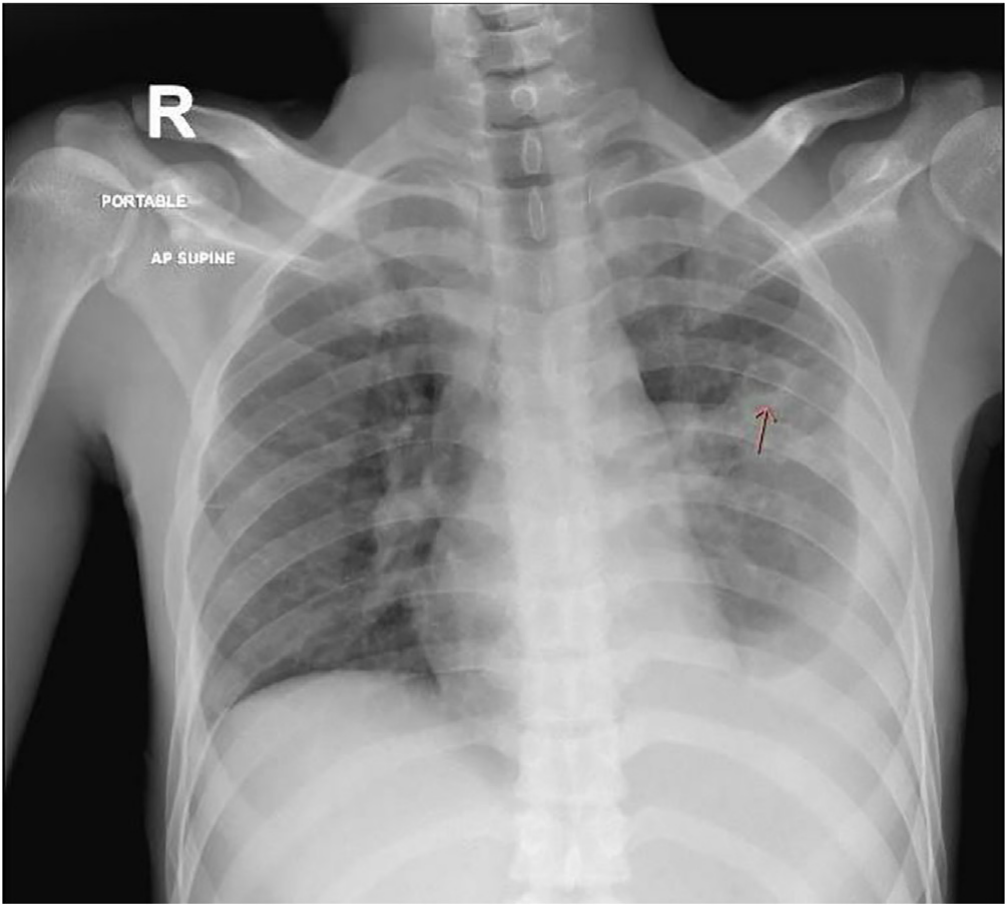
Bilateral infiltrates with left middle zone cavitary lesion and left sided pleural effusion.

The most common symptoms were cough (83.3 %), followed by fever (50 %), myalgias (33.3 %), headache (16.6 %) and fatigue (16.6 %). None of our patients reported diarrhea, vomiting, loss of smell, or taste. The main B-type symptom was weight-loss (83.3 %). Lab results included a mean neutrophil count 7.6*10^3^ cells/mm^3^, lymphocyte count 1.3*10^3^ cells/mm^3^ and ALT 28.1 U/l. Neutrophil/lymphocyte ratio (NLR) was more than 3.13 in all patients. Screening for hepatitis B, hepatitis C, and human immunodeficiency viruses (HIV) was negative in all patients. All patients had cavitary lesions identified on imaging (Fig. 1, Fig. 2, Fig. 3, Fig. 4, Fig. 5, Fig. 6). Patient 6 also had pleural effusion which turned out to be lymphocytic exudative with a positive nucleic acid amplification test for TB (Fig. 1e).

### Diagnosis

The diagnosis of pulmonary TB in patients 1–6 was initially based on a positive sputum smear for acid-fast bacilli (AFB). Later, the TB culture from sputum sample turned out to be positive in all patients. All patients were smear-negative by 6 weeks of treatment with median of 3 weeks **(**Table 2**)**.

**Table 2:** Diagnosis.

	Patient 1	Patient 2	Patient 3	Patient 4	Patient 5	Patient 6
IGRA for TB	Negative	Indeterminate	Indeterminate	Indeterminate	Positive	Positive
AFB smear status	Positive from sputum	Positive from sputum	Positive from sputum	Positive from sputum	Positive from sputum	Positive from sputum
AFB culture status	Positive from sputum	Positive from sputum	Positive from sputum	Positive from sputum	Positive from sputum	Positive from sputum
TB Diagnosis	Pulmonary TB	Pulmonary TB	Pulmonary TB	Pulmonary TB	Pulmonary TB	Pulmonary and pleural TB
SARS-CoV-2 RT-PCR (Nasopharyngeal Swab)	Positive	Positive	Positive	Positive	Positive	Positive
Number of days for negative SARS-CoV-2 RT-PCR	42	42	14	72	28	21
Number of days for negative AFB smear/TB PCR	42	14	28	21	21	14
New Diagnosis during hospitalization	Pre-Diabetes	Diabetes Mellites	Anemia	Pre-diabetes	Anemia	None

(IGRA: Interferon Gamma Release Assay, AFB: Acid Fast Bacillus, TB: Tuberculosis, SARS-CoV-2: Severe Acute Respiratory Syndrome Coronavirus-2, RT-PCR: Reverse Transcriptase-Polymerase Chain Reaction).

All patients tested positive for SARS-CoV-2 RNA by RT-PCR of the nasopharyngeal swab. Follow-up COVID-19 oropharyngeal and nasopharyngeal swabs were initially repeated two weeks from diagnosis and then weekly until negative. The clearance of virus was variable, but two patients had positive SARS-CoV-2 RT-PCR beyond four weeks **(**Table 2**).**

### Treatment

All patients received five days of Ceftriaxone, Azithromycin, and Hydroxychloroquine for COVID-19 pneumonia, according to the local COVID-19 management guidelines at that time. All patients also received first-line anti-tuberculous therapy (ATT) consisting of Isoniazid, Rifampicin, Pyrazinamide, and Ethambutol with Pyridoxine.

### Prognosis and outcome

All patients admitted from the emergency improved with treatment and were discharged in a stable condition from the hospital.

## Discussion

There is a paucity of data reported on TB and SARS-CoV-2 coinfection, which is likely due to under-reporting. However, missed diagnosis, early mortality, and limited testing facilities for SARS-CoV-2 may also be responsible [5]. None of our patients had a known history of direct exposure to TB; however, all of them belonged to areas where TB is endemic i.e. South-Asian descent. The exact mechanism underlying COVID-19 and TB coinfection is unknown. An interplay of various interleukins (IL) triggered by inflammatory response to viral infection that affect T-cell Immune response is an argument found in the literature [6]. Type-I interferon (IFN) has antiviral properties, but detrimental effects of inappropriate or mistimed type-I IFN response in viral infections also exist that may promote susceptibility to TB infection [7].

Other factors, such as dysregulated glucose metabolism, predispose patients to an excessive uncontrolled inflammatory response, which leads to a higher risk of developing COVID-19 pneumonia and rapid disease progression [8]. Additionally, DM also increases the risk of developing active tuberculosis infection by two to four folds [9]. Only one of our patients had established diagnosis of DM. However, 66.6 % of our patients had impaired glucose metabolism with an average HbA1c of 6.48. It is also important to note that a population with low socioeconomic status may carry a higher risk of exposure and disease spread [10,11], as seen in our case series.

In terms of risk stratification, Ai-Ping Yang et al. have studied neutrophil to lymphocyte ratio (NLR) and age as an independent biomarker for poor clinical outcome in COVID-19 patients [12]. All our patients had an NLR greater than 3.3 with a mean ferritin level of 1686 ug/L and CRP 98.7 mg/L denoting at least moderate disease severity in an otherwise healthy young population (Table 2). The limitation to interpreting these parameters is coinfection with TB that makes it difficult to assess COVID-19 related disease severity.

Detecting patients with this coinfection has implications for patient management. Cohorting COVID-19 patients with patients who have undiagnosed MTB may lead to its nosocomial transmission. COVID-19 patients with pulmonary TB coinfection will require airborne isolation, even if they do not receive invasive or non-invasive mechanical ventilation. This may affect the length of stay and pose a challenge in a limited resource setting.

## Conclusion

Coinfection with COVID-19 and Mycobacterium tuberculosis may alter disease course and management. A high index of suspicion is required for detection of this coinfection especially in population at high risk for TB or belonging to endemic areas. Coinfection may not correlate with worse outcomes if recognized early.

## Author contributions

The first three authors contributed equally to this article. ZY conceived the topic idea, identified the cases, and managed some of the cases. Additionally, he performed the initial literature review, data analysis, and wrote the initial manuscript. AAK assisted in the literature review and wrote up the manuscript. HAC critically reviewed and revised the final manuscript. KM, MUK and MFHM assisted in analyzing the cases and critically revising the manuscript. JB and SAK were involved in managing the cases and reviewed the data as infectious disease experts. All authors approved the final version for submission.

Consent

Written informed consent was obtained from the patient for publication of this case report. A copy of the written consent is available for review by the Editor-in-Chief of this journal on request.

## CRediT authorship contribution statement

**Zohaib Yousaf:** Conceptualization, Methodology, Project administration, Writing - original draft. **Adeel A. Khan:** Writing - original draft. **Haseeb A. Chaudhary:** Writing - review & editing. **Kamran Mushtaq:** Writing - review & editing. **Jabeed Parengal:** Supervision. **Mohamad Aboukamar:** Supervision. **Muhammad Umair Khan:** Project administration. **Mouhand F.H. Mohamed:** Writing - review & editing.

## Declaration of Competing Interest

The authors declare no conflict of interest.
